# Controlling for localised spatio-temporal autocorrelation in long-term air pollution and health studies

**DOI:** 10.1177/0962280214527384

**Published:** 2014-12

**Authors:** Duncan Lee, Richard Mitchell

**Affiliations:** 1 School of Mathematics and Statistics, University of Glasgow, Glasgow, UK; 2 Institute for Health and Wellbeing, University of Glasgow, Glasgow, UK

**Keywords:** air pollution and health studies, Gaussian Markov random fields, spatio-temporal autocorrelation

## Abstract

Estimating the long-term health impact of air pollution using an ecological spatio-temporal study design is a challenging task, due to the presence of residual spatio-temporal autocorrelation in the health counts after adjusting for the covariate effects. This autocorrelation is commonly modelled by a set of random effects represented by a Gaussian Markov random field (GMRF) prior distribution, as part of a hierarchical Bayesian model. However, GMRF models typically assume the random effects are globally smooth in space and time, and thus are likely to be collinear to any spatially and temporally smooth covariates such as air pollution. Such collinearity leads to poor estimation performance of the estimated fixed effects, and motivated by this epidemiological problem, this paper proposes new GMRF methodology to allow for localised spatio-temporal smoothing. This means random effects that are either geographically or temporally adjacent are allowed to be autocorrelated or conditionally independent, which allows more flexible autocorrelation structures to be represented. This increased flexibility results in improved fixed effects estimation compared with global smoothing models, which is evidenced by our simulation study. The methodology is then applied to the motivating study investigating the long-term effects of air pollution on respiratory ill health in Greater Glasgow, Scotland between 2007 and 2011.

## 1 Introduction

Air pollution has both a financial and a public health impact. The Department for the Environment, Food and Rural Affairs (DEFRA) in the UK estimate that “in 2008 air pollution in the form of anthropogenic particulate matter (PM) alone was estimated to reduce average life expectancy in the UK by six months. Thereby imposing an estimated equivalent health cost of £19 billion”.^1^ These estimated effects are based on evidence from large numbers of epidemiological studies, which collectively quantify the impact of exposure over both the short and the long term. The impact of long-term exposure has typically been estimated using individual-level cohort studies,^2^ but due to the long-term follow-up required for the cohort, such studies are costly and time consuming to implement. Therefore the recent routine availability of small-area statistics has led to ecological spatio-temporal study designs also being used.^3–10^ These studies cannot assess the causal health effects of air pollution due to their ecological design, but as they are quick and inexpensive to implement they add to the body of evidence about the long-term health impact of air pollution.

Ecological spatio-temporal studies utilise data relating to *K* non-overlapping areal units for *T* consecutive time periods, which include population-level measurements of disease, pollution concentrations and other confounding factors such as socio-economic deprivation and demography. Poisson log-linear models are typically used to estimate the effects of air pollution on health, because the disease data often take the form of counts of the numbers of cases for each areal unit and time period. The simplest such model is a Poisson generalised linear model, where only the available covariates are used to explain the spatio-temporal pattern in the disease data. However, the residuals from such models typically display spatio-temporal autocorrelation, which violates the assumption of independence made in such models. This residual spatio-temporal autocorrelation could be caused by numerous factors, such as unmeasured confounding, neighbourhood effects (where subjects behaviour is influenced by neighbouring subjects), grouping effects (where subjects choose to be close to similar subjects) and the fact that disease counts in consecutive time periods come from largely the same susceptible population. Ignoring this autocorrelation using the naive Poisson model described above can adversely affect both the covariate effect estimates and the coverage probabilities of the corresponding confidence intervals, and models that adjust for this autocorrelation are required.

Purely spatial studies have accounted for this residual autocorrelation using many different approaches, including conditional autoregressive models,^6^ simultaneous autoregressive models^3^ and geographically weighted regression.^7^ However, only one study^10^ has allowed for such residual autocorrelation in a spatio-temporal study design, because other existing studies^4,5,9^ do not relate to contiguous areal units. The main mechanism for modelling this autocorrelation is via a set of random effects, whose prior distribution induces either spatial or spatio-temporal smoothness in their values. However, recent research^11,12^ in a purely spatial setting has shown the potential for collinearity between spatially smooth covariates such as air pollution and spatially smooth random effects, which may lead to variance inflation and poor fixed effects estimation.^13^ Furthermore, the residual spatial autocorrelation from a covariate only model may be too complex to be adequately represented by a set of globally smooth random effects, as some scales of global spatial variation will have been accounted for by covariates included in the model such as air pollution. Thus the unexplained residual spatial variation may be localised, with strong autocorrelation being observed between some pairs of geographically adjacent residuals while other pairs have very different values.

These problems are likely to carry-over to the spatio-temporal setting, and are illustrated by our motivating study of air pollution and health in Glasgow, Scotland, between 2007 and 2011. The data for this study are presented in Section 2, which also presents an initial analysis based on a Poisson generalised linear model which assumes the disease counts are independent conditional on the covariates. The results from this initial analysis illustrate the need for a random effects model that can account for localised residual spatio-temporal variation, and the goal of this paper is to develop such a model. Our approach builds on recent work on localised smoothing in a purely spatial context,^14,15^ and is the first paper to consider this problem in a spatio-temporal rather than a purely spatial domain. Our general approach is based on Gaussian Markov random field (GMRF) models, which are extended so that the spatio-temporal adjacency structure of the data is estimated rather than being fixed. This general approach allows random effects that are geographically or temporally adjacent to be autocorrelated or conditionally independent, corresponding to either smoothing or not smoothing their values. However, the price of this flexibility is a large increase in the number of partial correlation parameters, and a critique of the existing solutions to this problem in a purely spatial context is presented in Section 3. This Section also summarises the spatio-temporal random effect models commonly used in a disease data context, and provides the background for the methodology we propose in Section 4. A simulation study is presented in Section 5 to evidence the improved estimation performance of our proposed model compared with commonly used alternatives, and the results of the Greater Glasgow study are presented in Section 6. Finally, Section 7 discusses the implications of our findings and avenues for future work.

## 2 Study design and initial analysis

**Figure 1.**
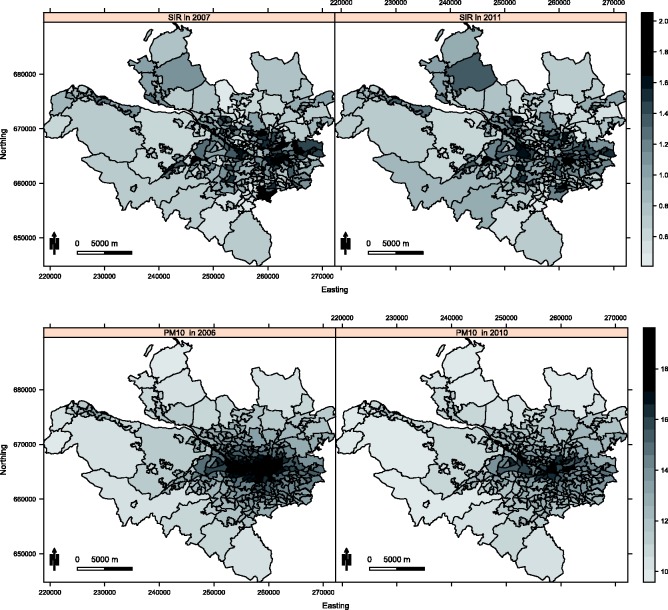
Maps displaying the standardised incidence ratio (SIR) for respiratory disease in 2007 (top left panel) and 2011 (top right panel) and the annual mean concentration for particles less than 10 µm (PM_10_) in 2006 (bottom left panel) and 2010 (bottom right panel).

The methodology developed in this paper is motivated by an epidemiological study investigating the health impact of long-term exposure to air pollution in the Greater Glasgow and Clyde Health Board in Scotland, for the five year period spanning 2007 to 2011. Glasgow is the largest city in Scotland, and the health board had a population of between 1,187,062 and 1,205,334 people during the study. The health board is split into *K* = 271 administrative units called intermediate geographies (IGs), which contained populations of between 2468 and 9517 people. The layout of the health board is presented in Figure 1, and the city of Glasgow is the set of small IGs in the east of the figure. The data are described below, and both the disease and confounder data are available from the Scottish Neighbourhood Statistics (SNS) database (http://www.sns.gov.uk), while the modelled pollution concentrations are available from the DEFRA (http://uk-air.defra.gov.uk/data/pcm-data).

### 2.1 Description of the data

The disease data are yearly counts of the numbers of people in each IG admitted to non-psychiatric and non-obstetric hospitals with a primary diagnosis of respiratory disease, which corresponds to the *International Classification of Disease tenth revision* (*ICD-10*) codes J00-J99 and R09.1. However, the set of IGs have different population sizes and demographic structures, so external standardisation was used to compute the expected numbers of disease cases in each area and year, using age and sex specific disease rates for the entire population. An exploratory estimate of disease risk is given by the standardised incidence ratio (SIR, observed cases/expected cases), which is displayed for the first and last years of the study in the top row of Figure 1. The figure shows that the risks are highest in the heavily deprived east end of Glasgow (east of the study region) and along the southern bank of the river Clyde (which starts as an estuary in the west and moves south east across the study region). While the main spatial pattern in disease risk has not changed over the five year period, there are IGs that exhibit elevated and reduced risks in 2011 compared with 2007, suggesting that the spatio-temporal correlation structure in these data is likely to be non-separable.

Ambient air pollution concentrations are measured at a network of locations across Scotland, details of which are available at the Scottish Air Quality website (http://www.scottishairquality.co.uk/). However, the network is not dense at the small-area scale required by this study, so instead we make use of modelled yearly average concentrations between 2006 and 2010 at a resolution of 1 km grid squares provided by the DEFRA. These modelled concentrations were computed using dispersion models and calibrated against the available monitoring data, and further details are available.^16^ The gridded data were converted to the IG scale by computing the median value within each IG, and modelled concentrations have previously been used in small-area air pollution and health studies.^6,8^ We note that the use of modelled concentrations is likely to result in exposure measurement error, but in addition to being used by the studies above, they are the only pollution data available at the high spatial resolution we require. We use concentrations lagged by one year relative to the disease data, because it ensures that the exposure occurred before disease onset. The pollutants we consider in this study are annual mean concentrations of carbon monoxide (CO, in mgm^−3^), nitrogen dioxide (NO_2_, in µgm^−3^) and particulate matter (PM), the latter being measured as particles less than 2.5 µm (PM_2.5_, in µgm^−3^) and 10 µm (PM_10_, in µgm^−3^) in diameter. The yearly average concentrations of PM_10_ in 2006 and 2010 are displayed in the bottom panel of Figure 1, which shows that the highest concentrations are in the centre of the city of Glasgow as expected. In addition, Table 1 summarises the data, and shows that pollution concentrations were generally highest at the start (2006) and again at the end (2010) of the study period, with lower values being observed between these two years.

**Table 1.** Summary of the spatio-temporal variation in the data between year 1 (2007) and year 5 (2011) of the study, presented as the mean (standard deviation) value across the study region.^a^

Variable	Year 1	Year 2	Year 3	Year 4	Year 5
Respiratory disease	75.3 (32.8)	81.0 (37.0)	78.1 (34.2)	78.4 (33.0)	83.2 (35.0)
CO (mgm^−3^)	0.19 (0.04)	0.19 (0.02)	0.20 (0.01)	0.18 (0.01)	0.22 (0.01)
NO_2_ (µgm^−3^)	17.4 (6.7)	15.7 (5.6)	15.2 (5.3)	15.4 (5.0)	17.0 (6.3)
PM_2.5_ (µgm^−3^)	8.2 (1.2)	5.8 (0.8)	6.9 (1.1)	7.4 (1.1)	9.0 (1.2)
PM_10_ (µgm^−3^)	13.9 (2.0)	12.3 (1.5)	10.9 (1.5)	11.5 (1.5)	13.0 (1.7)
Job seekers allowance (%)	2.9 (1.8)	3.2 (1.9)	4.8 (2.5)	5.0 (2.7)	5.1 (2.8)
House price (£, 000)	133.5 (55.0)	134.8 (53.4)	121.3 (49.6)	124.9 (56.4)	123.9 (58.1)
Ethnicity (%)	9.2 (13.0)	9.8 (13.3)	10.3 (13.6)	10.7 (13.7)	11.1 (14.1)

a Note, the pollution concentrations are lagged by one year relative to the disease data.

The main confounding factor in ecological health studies is socio-economic deprivation,^17^ and data on median property price and the percentage of people who are in receipt of job seekers allowance (JSA) are available. In the current study, where the response is a measure of respiratory ill health, the level of socio-economic deprivation in an IG is likely to be acting as a proxy measure of smoking prevalence, which is the major risk factor for respiratory health. Yearly smoking prevalence data for each IG are not available, and existing studies^18^ have shown area level measures of socio-economic deprivation to be good proxy measures. The other potential confounder considered in this study is a measure of ethnicity, because different rates of respiratory disease have been observed for people from different ethnic backgrounds.^19^ The only variable available to measure ethnicity is the percentage of school children living in each IG who are non-white, which we appreciate is imperfect as it does not differentiate between people of different non-white ethnicities.

### 2.2 A generalised linear model analysis

The study region is partitioned into k=1,…,K , (*K* = 271) IGs, and data for each unit are collected for t=1,…,T , (*T* = 5) consecutive years. The total number of respiratory hospitalisations in area *k* during time period *t* is denoted by *Y_kt_*, while *E_kt_* is the expected number of cases included as an offset term in the model to account for varying population sizes and demographic structures across the IGs. The vector of *p* covariates is denoted by $x_{\mathrm{kt}}^{\text{T}}=(x_{1\mathrm{kt}},\ldots ,x_{\mathrm{pkt}})$ , and is multiplied by a vector of regression parameters $\beta =(\beta_{1},\ldots ,\beta_{p})$ . The covariates include air pollution concentrations, indices of socio-economic deprivation and an intercept term. Initially, an overdispersed Poisson generalised linear model was fitted to the disease count data *Y_kt_*, which is given by

(1) $$Y_{\mathrm{kt}}|E_{\mathrm{kt}},R_{\mathrm{kt}}\sim \text{Poisson}(E_{\mathrm{kt}}R_{\mathrm{kt}})\text{for}k=1,\ldots ,K\text{and}t=1,\ldots ,T,\text{In}(R_{\mathrm{kt}})=x_{\mathrm{kt}}^{\text{T}}\beta$$

where *R_kt_* denotes the risk of disease in areal unit *k* during time period *t*. This model assumes the disease counts are independent conditional on the available covariates. The model contained the non-pollution covariates, and each of the four pollutants was included in separate models because of the potential for collinearity between them (pairwise correlations ranged between 0.67 and 0.89). The remaining three covariates all exhibited substantial or borderline substantial relationships with respiratory disease, and were thus retained in the model. The estimated covariate effects are displayed in Table 3, and are presented as relative risks for a one standard deviation increase in each covariates value. The table shows substantial health impacts of CO, NO_2_ and PM_10_, as their 95% confidence intervals do not contain the null risk of one. The size of the estimated relative risks is small compared to the effects of socio-economic deprivation, but as the population at risk is around 1.2 million the public health impact is sizeable.

**Figure 2.**
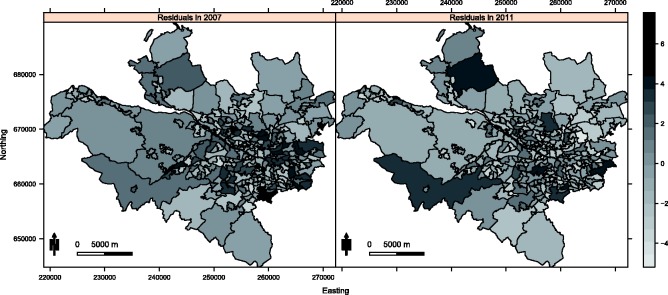
Maps displaying the residuals from the Poisson generalised linear model for 2007 and 2011.

To assess the appropriateness of this model the raw residuals were computed, and are displayed in Figure 2 for 2007 and 2011. The residuals exhibit substantial overdispersion with respect to the Poisson distribution, with estimated overdispersion parameters ranging between 4.39 and 4.92 depending on the pollutant included in the model. To assess the presence of residual spatial autocorrelation, a permutation test based on Moran's I statistic^20^ using 10,000 random permutations was conducted for each year of the study separately. Statistically significant spatial autocorrelation was observed for each year, with *p* values ranging between 0.0101 and 0.0000999. The space–time separability of the residuals was then assessed, by computing correlation coefficients between residual spatial surfaces for each year. The correlations ranged between 0.335 and 0.646, suggesting that this residual autocorrelation is non-separable in space and time, a result which is visually corroborated by Figure 2. The figure also shows that the residual spatial autocorrelation is localised in both years, with some pairs of adjacent areal units having similar values suggesting spatial smoothness, while others have very different values suggesting a lack of smoothness.

## 3 Spatio-temporal autocorrelation models

### 3.1 Existing models

Models that account for the spatio-temporal autocorrelation in the data extend equation (1) by adding in a set of random effects to the linear predictor, and the first level of such a model is given by

(2) $$Y_{\mathrm{kt}}|E_{\mathrm{kt}},R_{\mathrm{kt}}\sim \text{Poisson}(E_{\mathrm{kt}}R_{\mathrm{kt}})\text{for}k=1,\ldots ,K\text{and}t=1,\ldots ,T,\;\ln(R_{\mathrm{kt}})=x_{\mathrm{kt}}^{\text{T}}\beta +\varphi_{\mathrm{kt}}$$

Here the spatio-temporal pattern in the log risk is represented by covariates $x_{\mathrm{kt}}^{\text{T}}$ and a random effect $\varphi_{\mathrm{kt}}$ , the latter of which captures any residual spatio-temporal autocorrelation that is not accounted for by the covariates. Inference for this model is typically conducted in a Bayesian setting, with inference based on either Markov chain Monte Carlo (McMC) simulation or integrated Nested Laplace approximations^21^ (INLA). One of the first random effect models to capture spatio-temporal trends and autocorrelation in areal unit disease data was in the related field of disease mapping,^22^ and made the assumption that the spatial and temporal structure was separable. This means that the spatial pattern in the risk surface does not change over time, and their model was given by

(3) $$\varphi_{\mathrm{kt}}=\varphi_{k}^{\left(1\right)}+\varphi_{k}^{\left(2\right)}+\varphi_{t}^{\left(3\right)}+\varphi_{t}^{\left(4\right)}$$

$$\varphi_{k}^{\left(1\right)}|{\overset{\sim }{\varphi }}_{-k}^{\left(1\right)},\;U\sim \text{N}(\frac{\sum_{i=1}^{K}u_{k,i}\varphi_{i}^{\left(1\right)}}{\sum_{i=1}^{K}u_{k,i}},\frac{1}{\tau_{1}(\sum_{i=1}^{K}u_{k,i})}),$$

$$\varphi_{k}^{\left(2\right)}\sim \text{N}(0,\frac{1}{\tau_{2}}),$$

$$\varphi_{t}^{\left(3\right)}|\varphi_{t-1}^{\left(3\right)}\sim \text{N}(\varphi_{t-1}^{\left(3\right)},\frac{1}{\tau_{3}}),$$

$$\varphi_{t}^{\left(4\right)}\sim \text{N}(0,\frac{1}{\tau_{4}})$$

In this model, $\varphi_{k}^{\left(1\right)}+\varphi_{k}^{\left(2\right)}$ is the common spatial surface for all time points, and $\varphi_{t}^{\left(3\right)}+\varphi_{t}^{\left(4\right)}$ is the common temporal trend for all areal units, and each is modelled as a linear combination of autocorrelated ( $\varphi_{k}^{\left(1\right)},\varphi_{t}^{\left(3\right)}$ ) and independent ( $\varphi_{k}^{\left(2\right)},\varphi_{t}^{\left(4\right)}$ ) processes. The independent processes are modelled with zero-mean Gaussian prior distributions with a constant variance, while the temporally autocorrelated process $\varphi_{t}^{\left(3\right)}$ is assigned a first order random walk prior distribution. The spatially autocorrelated process is modelled by an intrinsic conditional autoregressive^23^ (ICAR) model, and the correlation is induced via a binary *K* × *K* spatial neighbourhood matrix *U* = (*u_k_*_,_*_i_*), where *u_k_*_,_*_i_* = 1 if areal units (*k, i*) share a common border (also denoted by k~i ) and is zero otherwise. The model is presented above in its full conditional distribution form $f(\varphi_{k}^{\left(1\right)}|{\overset{\sim }{\varphi }}_{-k}^{\left(1\right)})$ , where ${\overset{\sim }{\varphi }}_{-k}^{\left(1\right)}$ denotes the vector of random effects except that in area *k*. The expectation for this prior is the mean of the random effects in neighbouring (geographically adjacent) areal units, while the variance is inversely proportional to the number of neighbouring units. The prior distributions for all four sets of random effects are examples of GMRFs, and can be re-expressed as a zero-mean multivariate Gaussian distribution with a sparse and potentially singular precision matrix. The precision matrix for the ICAR model is given by $\tau_{1}Q(U)$ , where *Q*(*U*) is a singular matrix given by Q(U)=diag(U1)-U and **1** is a vector of ones. This model can be seen as a separable spatio-temporal extension to the purely spatial Besag-York-Mollié^23^ (BYM) model commonly used for modelling disease risk, which only includes the two spatial terms $\varphi_{k}^{\left(1\right)}+\varphi_{k}^{\left(2\right)}$ .

However, as illustrated in Section 2 the residual spatio-temporal autocorrelation in the Glasgow respiratory disease data is unlikely to be separable, and one of the first non-separable extensions to equation (3) added a fifth set of spatio-temporal random effects.^24^ This model was used in a disease mapping context with no covariates, where the spatio-temporal random effects were of direct interest. In contrast, in the ecological regression context considered here they are only included to account for residual spatio-temporal autocorrelation, and are not of direct interest. Therefore a simpler, in terms of the number of random effects terms, non-separable spatio-temporal extension of the BYM model is given by

(4) $$\varphi_{\mathrm{kt}}=\varphi_{\mathrm{kt}}^{\left(1\right)}+\varphi_{\mathrm{kt}}^{\left(2\right)}$$

**Figure:**
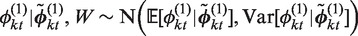

$$\varphi_{\mathrm{kt}}^{\left(2\right)}\sim \text{N}(0,\frac{1}{\tau_{2}})$$

where ${\overset{\sim }{\varphi }}_{\mathrm{kt}}^{\left(1\right)}$ denotes the vector of random effects for all time periods and areal units except $\varphi_{\mathrm{kt}}^{\left(1\right)}$ . This model combines autocorrelated ( $\varphi_{\mathrm{kt}}^{\left(1\right)}$ ) and independent ( $\varphi_{\mathrm{kt}}^{\left(2\right)}$ ) random effects, the latter again coming from independent zero-mean Gaussian distributions with a constant variance. The prior distribution for $\varphi_{\mathrm{kt}}^{\left(1\right)}$ is a spatio-temporal extension of the ICAR prior, where the spatio-temporal neighbourhood matrix is denoted by *W* and is given by

(5) $$W=(U^{\left(1\right)}V^{\left(1,2\right)}V^{\left(1,2\right)}U^{\left(2\right)}V^{\left(2,3\right)}V^{\left(2,3\right)}U^{\left(T-1\right)}V^{\left(T-1,T\right)}V^{\left(T-1,T\right)}U^{\left(T\right)})$$

Here $U^{\left(t\right)}=(u_{k,i}^{\left(t\right)})$ is a binary spatial neighbourhood matrix that determines the geographical adjacency between the *K* areal units during time period *t*, and is the mechanism by which correlation is induced between random effects relating to the same time period. Here, although not in the methodology proposed in the next section, *U*^(*t*)^ = *U* for all *t*. Similarly, $V^{\left(t,t+1\right)}=(v_{k,i}^{\left(t,t+1\right)})$ is a binary *K* × *K* temporal neighbourhood matrix, which is the mechanism by which correlation is induced between $\left(\varphi_{\mathrm{kt}}^{\left(1\right)},\varphi_{k(t+1)}^{\left(1\right)}\right)$ . Here we assume that the only temporal autocorrelation is between random effects from the same area one time period apart, which corresponds to $V^{\left(t,t+1\right)}=I$ for all *t*, where *I* is the identity matrix. Given this neighbourhood matrix the vector of spatially and temporally autocorrelated random effects $\varphi_{\mathrm{KT}\times 1}^{\left(1\right)}$ are modelled by a GMRF prior with a singular ICAR precision matrix τ_1_*Q*(*W*), where Q(W)=diag(W1)-W . The conditional expectation and variance for $\varphi_{\mathrm{kt}}^{\left(1\right)}|{\overset{\sim }{\varphi }}_{\mathrm{kt}}^{\left(1\right)}$ defined in equation (4) are given by:

**Figure:**
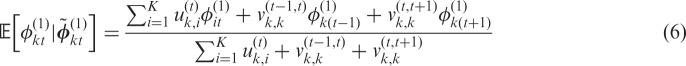

$$\text{Var}[\varphi_{\mathrm{kt}}^{\left(1\right)}|{\overset{\sim }{\varphi }}_{\mathrm{kt}}^{\left(1\right)}]=\frac{1}{\tau_{1}(\sum_{i=1}^{K}u_{k,i}^{\left(t\right)}+v_{k,k}^{\left(t-1,t\right)}+v_{k,k}^{\left(t,t+1\right)})}$$

with obvious modifications when *t* = 1,*T*. This model induces spatio-temporal smoothing by modelling the conditional expectation of $\varphi_{\mathrm{kt}}^{\left(1\right)}$ as the mean of the random effects in geographically adjacent areal units from the same time period and those from adjacent time periods relating to the same areal unit. Model (4) specifies two random effects $\left(\varphi_{\mathrm{kt}}^{\left(1\right)},\varphi_{\mathrm{kt}}^{\left(2\right)}\right)$ for each data point *Y_kt_*, and thus only their sum is reliably estimated from the data. Nevertheless, the spatial BYM model, of which this is an extension, is the most widely used model for capturing spatial autocorrelation in disease data, and in comparative studies it exhibits good overall performance.^25^

### 3.2 Global smoothing models and their limitations

Both the separable and non-separable models force global levels of spatial and temporal smoothness on the random effects, which are determined by the relative sizes of the precision parameters controlling the independent and autocorrelated processes. This is because the partial correlation between the autocorrelated random effects ( $\varphi_{\mathrm{kt}}^{\left(1\right)},\varphi_{\mathrm{jr}}^{\left(1\right)}$ ) is given by

(7) $$\text{Corr}[\varphi_{\mathrm{kt}}^{\left(1\right)},\varphi_{\mathrm{jr}}^{\left(1\right)}|{\overset{\sim }{\varphi }}_{-(\mathrm{kt},\mathrm{jr})}^{\left(1\right)}]=\frac{w}{\sqrt{\left(\sum_{i=1}^{K}u_{k,i}^{\left(t\right)}+v_{k,k}^{\left(t-1,t\right)}+v_{k,k}^{\left(t,t+1\right)})(\sum_{i=1}^{K}u_{j,i}^{\left(r\right)}+v_{j,j}^{\left(r-1,r\right)}+v_{j,j}^{\left(r,r+1\right)}\right)}}$$

where *w* is the element of the neighbourhood matrix *W* in equation (5) that corresponds to ( $\varphi_{\mathrm{kt}}^{\left(1\right)},\varphi_{\mathrm{jr}}^{\left(1\right)}$ ), and ${\overset{\sim }{\varphi }}_{-(\mathrm{kt},\mathrm{jr})}^{\left(1\right)}$ is the vector of all other random effects. Thus pairs of random effects that are either spatially or temporally adjacent are partially autocorrelated (their value of *w* = 1), while non-adjacent random effects are conditionally independent (their value of *w* = 0) given the remaining random effects. This suggests that if there is substantial spatio-temporal autocorrelation in the residuals that is if τ_1_ is relatively small compared to τ_2_, then all pairs of spatially or temporally adjacent random effects will be correlated. Therefore, these models are unable to capture localised residual spatio-temporal autocorrelation, which evolves smoothly in space and time between some pairs of adjacent data points but not between others.

A small number of papers have proposed localised smoothing approaches using GMRF priors in a purely spatial context, but no work has been done to extend these ideas to the spatio-temporal domain. The majority of the purely spatial approaches have treated the elements of the spatial adjacency matrix *U* corresponding to adjacent areal units as binary random quantities, rather than being fixed equal to one. Equation (7) shows that estimating an element of the adjacency matrix equal to one induces correlation between the random effects and smoothes their values towards each other, where as if it is estimated as zero then they are conditionally independent and no such spatial smoothing is enforced. Methods have been proposed for estimating the elements in *U* using measures quantifying the dissimilarity between pairs of areal units,^26^ but their approach was aimed at a disease mapping context, where as in the ecological regression context considered here covariate information would be included in the regression model. Therefore an iterative algorithmic approach has also been proposed,^14^ which deterministically updated the spatial adjacency matrix *U* based on the joint posterior distribution of the remaining model parameters. Finally, data on disease risk prior to the study period have been used to elicit a set of candidate spatial structures for the random effects,^15^ which are then used as the elements in a discrete prior distribution for *U* in an extended hierarchical model.

## 4 Methodology

The localised spatio-temporal smoothing model we propose follows the general approach of the existing literature in a purely spatial domain,^26,14,15^ and treats the elements of *W* relating to spatially or temporally adjacent observations as binary quantities to be estimated rather than fixed equal to one. From equation (7) it is clear that this allows adjacent random effects to be autocorrelated (if the element w∈W equals one) or conditionally independent (if the element w∈W equals zero), which either induces or does not induce smoothing between their values. Collectively denote the set of neighbourhood relations in *W* that correspond to spatially or temporally adjacent areal units by

**Figure:**
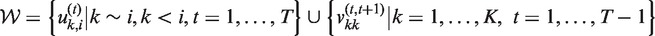

which in the global smoothing model (4) are all fixed at one. The major challenge in treating these as binary quantities to be estimated is overparameterisation, because the number of elements in 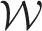 is large. For example, in the Greater Glasgow study there are 1355 data points (*K* = 271 and *T* = 5), while 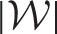 equals 4644. Therefore we do not treat each element in a Bayesian manner with a Bernoulli prior distribution, but instead propose an algorithm that iteratively re-estimates the localised spatio-temporal structure in the random effects until a convergence criterion is satisfied. The drawback of our proposed approach is that only a final estimate of each 
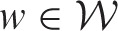
 is provided, where as the P(w=1) is not. This ignores the inherent uncertainty in 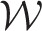 in the estimation process which we acknowledge is a limitation of our method, but it does enable localised smoothing to be undertaken without the need for additional data unlike existing approaches.^26,15^ The next section proposes a new random effects model based on a fixed neighbourhood structure 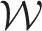 , while the iterative estimation algorithm is presented in Section 3.2. The model proposed here is an extension of the non-separable BYM model given by equation (4), because as has already been discussed the Glasgow data are likely to exhibit non-separable residual structure. However, separable residual spatio-temporal autocorrelation is possible in some applications, so the Supplementary material describes the analogous local smoothing extension of the separable BYM model (3).

### 4.1 A random effects model based on a fixed 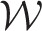


The model we propose combines equation (2) with a single *KT* × 1 vector of spatio-temporal random effects $\varphi =(\varphi_{11},\ldots ,\varphi_{\mathrm{KT}})$ , which contrasts with equation (4) which has separate spatial and non-spatial components. Thus our model does not suffer from the identifiability problems caused by modelling each data point with two random effects. The intrinsic CAR model given by equation (6) is inappropriate in this localised smoothing context, because as 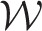 is a set of binary random quantities it is possible that $\sum_{i=1}^{K}u_{k,i}^{\left(t\right)}+v_{k,k}^{\left(t-1,t\right)}+v_{k,k}^{\left(t,t+1\right)}=0$ , for some areal unit and time period, leading to an infinite variance. Therefore we extend the LCAR prior^15^ into the spatio-temporal setting, by considering an extended vector of random effects $\overset{\sim }{\varphi }=(\varphi ,\varphi_{*})$ . Here $\varphi_{*}$ is a global random effect that is potentially common to all areal units and time periods, and its inclusion prevents the infinite variance problem described above. The extended (KT+1)×(KT+1) neighbourhood matrix for $\overset{\sim }{\varphi }$ based on a fixed *W*, the matrix equivalent of 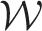 , is given by

(8) $$\overset{\sim }{W}=[Ww_{*}w_{*}^{\text{T}}0]$$

where $w_{*}^{\text{T}}=(w_{11,*},\ldots ,w_{\mathrm{KT},*})$ is a *KT* × 1 vector. The element $w_{\mathrm{kt},*}$ of $w_{*}$ controls the adjacency relationship between the global random effect $\varphi_{*}$ and $\varphi_{\mathrm{kt}}$ , and is given by

(9) $$w_{\mathrm{kt},*}=\text{I}[(1-v_{k,k}^{\left(t-1,t\right)})+(1-v_{k,k}^{\left(t,t+1\right)})+\sum_{i\sim k}(1-u_{k,i}^{\left(t\right)})>0]$$

Here I[.] denotes an indicator function, so that $w_{\mathrm{kt},*}=1$ if at least one of the above neighbourhood relations $\left(v_{k,k}^{\left(t-1,t\right)},v_{k,k}^{\left(t,t+1\right)},\{u_{k,i}^{\left(t\right)}|i\sim k\}\right)$ for areal unit *k* and time period *t* has been estimated as zero. Otherwise $w_{\mathrm{kt},*}=0$ , and obvious simplifications are made when *t* = 1 or *t* = *T*. Based on this extended neighbourhood matrix, we model $\overset{\sim }{\varphi }$ with a zero-mean GMRF with an ICAR precision matrix $\tau Q(\overset{\sim }{W})$ , where $Q(\overset{\sim }{W})=\text{diag}(\overset{\sim }{W}1)-\overset{\sim }{W}$ . The Gaussian full conditional distributions corresponding to this extended intrinsic model have expectation and variance given by

**Figure:**
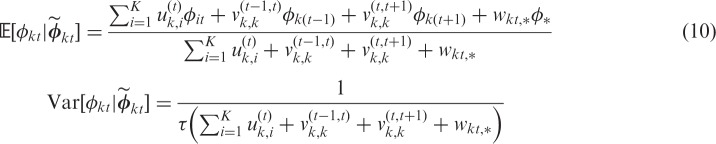

$$\text{Var}[\varphi_{\mathrm{kt}}|{\overset{\sim }{\varphi }}_{\mathrm{kt}}]=\frac{1}{\tau (\sum_{i=1}^{K}u_{k,i}^{\left(t\right)}+v_{k,k}^{\left(t-1,t\right)}+v_{k,k}^{\left(t,t+1\right)}+w_{\mathrm{kt},*})}$$

The conditional expectation is a weighted average of the global random effect $\varphi_{*}$ and the random effects in neighbouring spatial and temporal units, with the weights being determined by the current value of 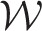 . This model can represent both spatio-temporal smoothing extremes, because if all elements in 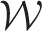 are estimated as one then the model is equivalent to just having the autocorrelated random effects $\varphi^{\left(1\right)}$ in equation (4), while if all the elements in 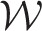 equal zero then the model is equivalent to just having the independent random effects $\varphi^{\left(2\right)}$ in equation (4). The full Bayesian hierarchical model we propose conditional on a fixed 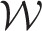 is given by

(11) $$Y_{\mathrm{kt}}|E_{\mathrm{kt}},R_{\mathrm{kt}}\sim \text{Poisson}(E_{\mathrm{kt}}R_{\mathrm{kt}})\text{for}k=1,\ldots ,K\text{and}t=1,\ldots ,T,\text{ln}(R_{\mathrm{kt}})=x_{\mathrm{kt}}^{\text{T}}\beta +\varphi_{\mathrm{kt}}$$

$$\beta_{i}\sim \text{N}(0,1000)\text{for}i=1,\ldots ,p$$

$$\overset{\sim }{\varphi }\sim \text{N}(0,\text{Prec}[\tau Q(\overset{\sim }{W})])$$

τ~Gamma(1,0.0005)

### 4.2 Estimation algorithm for localised spatio-temporal smoothing

The parameters $\Theta =(\beta ,\overset{\sim }{\varphi },\tau )$ are estimated via an iterative algorithm, which extends the algorithm proposed by Lee and Mitchell^14^ into the spatio-temporal domain. The algorithm cycles between updating Θ and 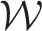 conditional on the other in turn until a termination criterion is met. Conditional on 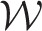 model (11) is fully Bayesian, and inference is implemented using INLA rather than McMC simulation for reasons of computational speed. This is because the estimation algorithm proposed here iteratively re-fits the model based on different neighbourhood structures 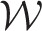 , which would make an McMC approach to estimation computationally prohibitive. In contrast, 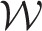 is updated deterministically at each iteration of the algorithm based on the current posterior distribution of Θ. Consider an element 
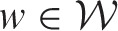
 , which determines the partial correlation between two spatially or temporally adjacent random effects. Its value is updated based on the posterior distribution of Θ using the following deterministic rule.

- Set *w* = 0 if the marginal 95% posterior credible intervals for the two random effects do not overlap, because there is evidence that they are substantially different.
- Set *w* = 1, if the marginal 95% posterior credible intervals for the two random effects do overlap, because there is no substantial difference between them.

This approach induces spatio-temporal smoothing between the next estimates of spatially or temporally adjacent random effects if the current estimates are similar, whereas no such smoothing is enforced if the current estimates are substantially different. The full iterative estimation algorithm proposed here is given below.

Algorithm

1: Estimate a starting posterior distribution 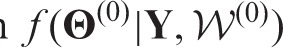 for Θ using INLA. For this initial model the random effects are assumed to be independent.

2: Iterate the following two steps for $l=1,2,\ldots ,l^{*}$ , until one of the two termination conditions for 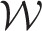 , outlined in step 3, are met.

 a: Estimate 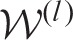 deterministically from the current posterior distribution 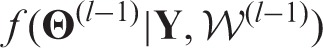 . Set 
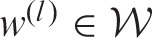
 equal to one if the marginal 95% posterior credible intervals for the two corresponding random effects overlap. Otherwise, set 
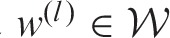
 equal to zero.

 b: Estimate the posterior distribution 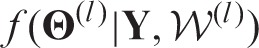 using INLA.

3: After l^*^ iterations one of the following two termination conditions will apply.

 **Case 1:** The sequence of 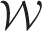 estimates is such that 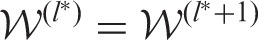 , which is the estimated neighbourhood structure 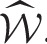 .

 **Case 2:** The sequence of 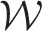 estimates forms a cycle of *k* different states 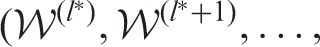

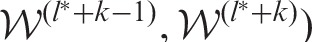
 , where 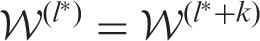 . In this case, the estimated neighbourhood structure 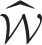 is the value from the cycle of *k* states that has the minimal level of residual spatio-temporal autocorrelation, as measured by the absolute value of Moran's I statistic.

When one of these termination conditions has been met 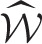 is the estimated spatio-temporal structure of the random effects, and Θ is summarised by the posterior distribution 
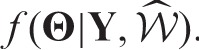
 .

The algorithm is initialised by assuming the random effects are independent, because this does not enforce any initial spatio-temporal smoothing constraints on the random effects surface. One of the two termination conditions outlined in the algorithm is guaranteed to apply after a sufficiently large number of iterations l*, because each 
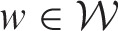
 is binary and hence the sample space for 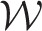 is large but finite. In practice however, the algorithm almost always terminates under case 1 in a small number of iterations.

## 5 Simulation study

This section presents a simulation study, which compares the performance of an overdispersed Poisson generalised linear model (1) with the global BYM model (4) and localised smoothing model (11) that account for non-separable spatio-temporal autocorrelation. In addition, the separable global BYM model (3) and a localised extension of it (described in the Supplementary material) are also compared, to see the impact that specifying an inappropriate autocorrelation structure has on fixed effects estimates.

### 5.1 Study design

Simulated data are generated for the 271 areal units that comprise the Greater Glasgow and Clyde health board for 2007–2011, which is the study region and time frame for our motivating study. Disease counts are generated from model (2), where the expected numbers *E_kt_* are uniform random draws from one of the following three intervals: (i) [10, 25], (ii) [50, 100] and (iii) [150, 200]. These values allow us to assess the robustness of our methodology when applied to disease data with different prevalences. The log risk surface is generated from a linear combination of covariates and random effects, the former including PM (PM_10_) and the other three covariates used in the Glasgow study. The regression parameters for the non-pollution covariates were fixed at the estimates obtained from the motivating study, where as the relative risk associated with a one standard deviation increase in PM_10_ was fixed at 1.03 to be similar to both this and existing studies.

**Figure 3.**
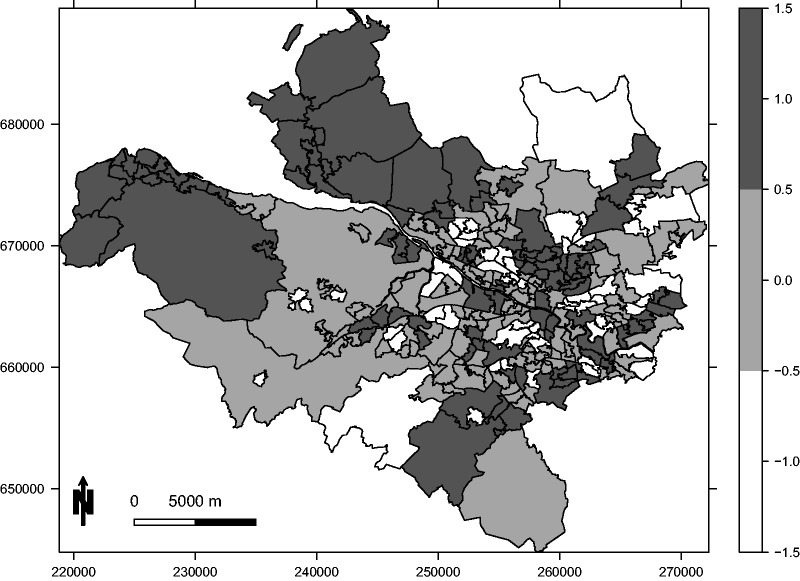
A map showing the piecewise constant mean function (with possible values {−1, 0, 1}) for the random effects that generate localised spatial autocorrelation in the first year of the simulation study.

The random effects are included to induce residual spatio-temporal autocorrelation into the log risk surface, which mimics its presence in the real data study. They are generated from a multivariate Gaussian distribution with a piecewise constant mean and a spatially and temporally smooth precision matrix, the latter being based on the neighbourhood matrix (5). Localised spatio-temporal structure is induced by the piecewise constant mean, which for the first year of the study follows the template shown in Figure 3. This template only has three distinct values {−1, 0, 1}, which are multiplied by a constant *M* to obtain the mean surface. Values of *M* = 0.5 and *M* = 1 are considered in this study, which respectively correspond to small and large localised structure. Finally, we consider scenarios where the random effects are separable and non-separable, with the former being achieved by using the same piecewise constant mean (given by Figure 3) for each year of the study. In contrast, non-separable random effects were generated by allowing the piecewise constant mean spatial surface given by Figure 3 to evolve each year, with the restriction that it still consisted of a series of spatial clusters.

### 5.2 Results

**Figure 4.**
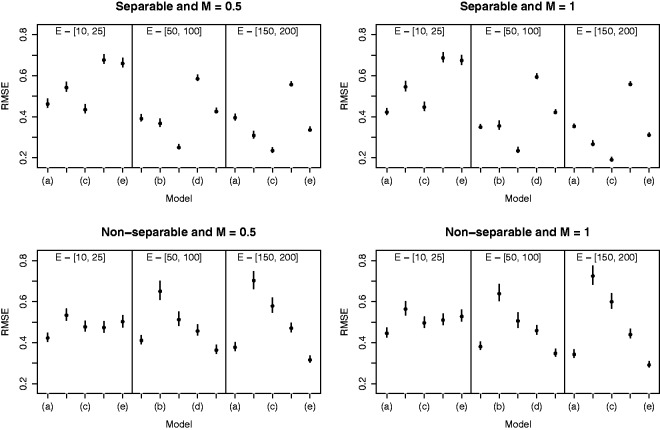
Root mean square error (RMSE) for the estimated regression parameter *β* for different values of *M* and **E** in the presence of either separable or non-separable residual spatio-temporal autocorrelation. In each case, the dot represents the RMSE while the vertical line is a bootstrapped 95% uncertainty interval. The models are: (a) Poisson generalised linear model (GLM), (b) Besag-York-Mollié (BYM) separable, (c) localised separable, (d) BYM non-separable and (e) localised non-separable.

Five hundred data sets are generated under each of 12 different scenarios, which include all pairwise combinations of: (i) *M* = 0.5, 1 (strength of the localised structure); (ii) $E_{\mathrm{kt}}\in [10,25],[50,100],[150,200]$ (disease prevalence); and (iii) separable and non-separable spatio-temporal autocorrelation in the data. The five models described at the start of this section are applied to each simulated data set, which are labelled as: (a) Poisson generalised linear model (GLM), (b) BYM separable, (c) localised separable, (d) BYM non-separable and (e) localised non-separable. The results are displayed in Figure 4 and Table 2, which respectively display the root mean square error (RMSE) of the estimated regression coefficient for PM_10_ and the coverage probability of its 95% uncertainty interval. We note that coverage probability is a frequentist concept, but it does enable us to quantify the appropriateness of the uncertainty intervals from each model.

**Table 2.** Coverage probabilities for the estimated regression parameter *β* for PM_10_ from each of the following models: (a) Poisson GLM, (b) BYM separable, (c) localised separable, (d) BYM non-separable, (e) localised non-separable.^a^

Coverage probability (%)				
*M*	**E**	(a)	(b)	(c)	(d)	(e)
Separable						
0.5	[10, 25]	84.0	95.6	95.6	94.2	94.8
0.5	[50, 100]	82.6	94.0	97.2	97.4	95.2
0.5	[150, 200]	78.4	89.4	91.4	99.6	87.2
1	[10, 25]	90.6	94.2	94.8	92.8	92.6
1	[50, 100]	92.4	94.2	97.4	97.8	96.2
1	[150, 200]	91.4	94.6	96.6	99.6	94.8
Non-separable						
0.5	[10, 25]	88.0	94.4	93.8	95.4	96.6
0.5	[50, 100]	81.4	73.6	75.2	95.0	96.8
0.5	[150, 200]	82.0	56.0	58.0	94.2	90.4
1	[10, 25]	87.0	92.8	94.0	94.6	96.6
1	[50, 100]	87.6	72.8	73.4	93.6	95.2
1	[150, 200]	86.0	55.4	57.4	95.6	93.2

a The top half of the table corresponds to data containing separable spatio-temporal autocorrelation, while the bottom half has non-separable structure. In both cases, the results are presented for different levels of localised structure (via *M*) and different disease prevalences (via **E**).

In Figure 4, the RMSE values are represented by a black dot for each scenario and model, while the vertical lines are bootstrapped 95% uncertainty intervals based on 1000 bootstrapped samples. The figure presents three main findings. Firstly, both separable and non-separable localised smoothing models generally outperform their BYM global smoothing counterparts even when the magnitude of the localised structure is small, exhibiting RMSE values that are substantially lower (the uncertainty intervals rarely overlap). These findings are consistent for diseases with moderate to large prevalences ( $E_{\mathrm{kt}}\in [50,100],[150,200]$ ), where as for rare diseases ( $E_{\mathrm{kt}}\in [10,25]$ ) the results are less clear cut. This latter point is especially true when the residual spatio-temporal structure is non-separable, where all models exhibit similar RMSE values. This is because for rare diseases the mean values $E_{\mathrm{kt}}R_{\mathrm{kt}}$ are small, and hence any underlying spatio-temporal pattern is overwhelmed by Poisson noise. The second finding from this study is that fitting an in-appropriate GMRF model results in poor fixed effects estimation performance, as if the spatio-temporal autocorrelation is separable then the separable models generally outperform their non-separable counterparts. Similarly the converse is also true. The third finding from this study is that the naive Poisson generalised linear model does not necessarily perform badly in terms of parameter estimation, as its RMSE values are in the most part in keeping with those from the other models. However, Table 2 shows that it does perform badly in terms of coverage probabilities, with coverages ranging between 78.4% and 92.4% across the range of scenarios considered here. In contrast, all of the GMRF models exhibit close to their nominal 95% coverage levels, with the exceptions being when either the BYM or localised separable models are applied to data with non-separable residual structure.

### 5.3 Results – algorithm convergence

The empirical convergence properties of the localised smoothing algorithm show that it terminates under case 1 in 93.5% of cases for the separable model and 99.9% of cases for the non-separable model. For both models the median number of iterations taken to converge is 4, which taken with the above result suggests that the algorithm typically converges quickly to a final value of 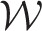 .

## 6 Glasgow study

The non-separable localised spatio-temporal smoothing model (11) proposed in Section 4 is now applied to the data from the Greater Glasgow study presented in Section 2, together with the non-separable global BYM smoothing model given by equations (2) and (4). We have not applied the separable variants of these two models to the Glasgow data because the initial analysis in Section 2 suggests that the residuals exhibit highly non-separable structure.

### 6.1 Model comparison

The overall fit of both non-separable models to the Glasgow respiratory data was compared using a number of different metrics, including the Deviance Information Criterion^27^ (DIC), the Conditional Predictive Ordinate (CPO), and the extent to which they adequately controlled for the spatio-temporal autocorrelation in the data. Both models have successfully accounted for this autocorrelation, as evidenced by Moran's I permutation tests on the residuals for each year yielding non-significant *p* values. The DIC for the BYM global smoothing model is 10,556 compared with 10,430 for the localised model, suggesting an improvement in in-sample fit to the data for the localised model proposed here. Conversely, the CPO for each observation is its predictive distribution conditional on the remainder of the data, that is $f(Y_{\mathrm{kt}}|Y_{-\mathrm{kt}})$ , where $Y_{-\mathrm{kt}}$ denotes the vector of observations except *Y_kt_*. It is thus a predictive measure of model fit, with larger values indicating a better fit to the data. Computing the mean CPO over all observations gives values of 0.0293 for the BYM global model and 0.0309 for the local smoothing model, indicating a small improvement in fit of the latter. The CPO is a local predictive goodness of fit measure, and the local smoothing model exhibited an improved fit compared to the BYM global model for 66% of data points (the global model exhibited an improved fit for 29% of data points). Thus in terms of both in-sample and predictive model fit the local smoothing model provides a small improvement on the BYM global model. In terms of the localised spatio-temporal autocorrelation in the data, the localised smoothing model has estimated 336 pairs of spatially or temporally adjacent random effects (elements in 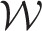 ) as being conditionally independent, which is 7.2% of the total number of adjacencies in the study region.

### 6.2 Estimated covariate effects

Estimated covariate effects and 95% credible intervals from the two models are displayed in Table 3, together with the initial results from a Poisson generalised linear model. In all cases, the results are presented as relative risks, for a one standard deviation increase in each covariates value. The table shows substantial differences in the estimated pollution effects between the three models, with the largest difference being for NO_2_ whose relative risk ranges between 1.5% and 8.8%. The pollution risks estimated from the Poisson generalised linear model are very large compared with those from the GMRF models, and are much larger than the risks observed in the existing literature.^5,6^ The results from the two GMRF models also exhibit substantial variation, as the estimate from the localised model is more than double that from the BYM global model for NO_2_, and is 28% larger for PM_10_. The results from the simulation study suggest that the estimates from the localised model are likely to be the most accurate, and based on these results increases in NO_2_ (by 5.78 µgm^−3^) and PM_10_ (by 1.96 µgm^−3^) are associated with increased health risks of 3.4% and 5.3%, respectively. In contrast, the effects of CO and PM_2.5_ are close to the null risk of one. The estimated effects from the BYM global smoothing model typically show attenuation compared with those from the localised model, which may be due to the effects of collinearity between the random effects and the pollution covariate.^13^ Finally, the two measures of socio-economic deprivation show substantial effects on the response, with an increased risk of 21.2% for JSA and a decreased risk of 10.1% for median house price. Finally, after the inclusion of the random effects, the ethnicity variable shows no substantial effect on respiratory disease risk.

**Table 3.** Estimated covariate effects and 95% credible intervals from the Poisson generalised linear model (1), the non-separable global BYM model (4) and the non-separable localised smoothing model (11).^a^

Spatio-temporal autocorrelation models			
Covariate	Poisson GLM	BYM	Localised
CO	1.026 (1.011, 1.042)	1.001 (0.980, 1.023)	1.008 (0.989, 1.027)
NO_2_	1.088 (1.071, 1.104)	1.015 (0.988, 1.042)	1.034 (1.011, 1.057)
PM_2.5_	1.012 (0.997, 1.027)	0.991 (0.969, 1.013)	0.994 (0.975, 1.013)
PM_10_	1.091 (1.076, 1.107)	1.038 (1.013, 1.062)	1.053 (1.032, 1.075)
JSA	1.169 (1.150, 1.188)	1.222 (1.199, 1.246)	1.212 (1.191, 1.235)
House price	0.860 (0.843, 0.877)	0.904 (0.886, 0.921)	0.899 (0.882, 0.916)
Ethnicity	0.986 (0.972, 1.001)	0.993 (0.972, 1.015)	0.993 (0.974, 1.012)

a The results are presented as relative risks, for a one standard deviation increase in each covariates value, which are: CO – 0.024, NO_2_ – 5.87, PM_2.5_ – 1.56, PM_10_ – 1.96, job seekers allowance (JSA) – 2.57%, house price – £ 54,800 and ethnicity – 13.5%.

## 7 Discussion

This paper has proposed a new GMRF model for capturing localised spatio-temporal autocorrelation in areal unit health data, which combines existing methods^14,15^ and extends both into the spatio-temporal domain. Models for both separable and non-separable spatio-temporal autocorrelation have been proposed, and have been shown by simulation to outperform their global smoothing counterparts when the magnitude of the localised autocorrelation is either small or large. The increased flexibility offered by these methods naturally comes at the cost of a large increase in the number of parameters to estimate, and an estimation algorithm is proposed which iteratively updates the structure of the spatio-temporal autocorrelation conditional on the remaining parameters until a convergence criterion is reached. The key advantage of this and other localised smoothing approaches is that temporally or spatially adjacent random effects are allowed to be autocorrelated or conditionally independent, which does not enforce smoothing between their values if the data suggest such smoothing is inappropriate. However, the downside of localised smoothing approaches is their increased complexity in terms of the numbers of parameters, and the main drawback of our solution to this problem is that the uncertainty in 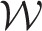 is ignored in the estimation process.

The main message from the simulation study is that the estimation of regression parameters for spatially and temporally smooth covariates is difficult, due to the presence of spatio-temporal autocorrelation in the data that is unexplained by the covariates. This autocorrelation is typically modelled by a set of random effects, and our study has shown that enforcing an inappropriate spatio-temporal structure on these effects is likely to lead to poor fixed effects estimation. In fact, ignoring this autocorrelation can lead to better fixed effects in a RMSE sense than modelling it with an inappropriate spatio-temporal structure, although such an approach does lead to 95% confidence intervals that are too narrow in terms of their coverage probabilities. However, models including spatio-temporal random effects are not without problems, as they have the potential for collinearity between the covariates and the random effects. The impact of this collinearity has previously been investigated in a purely spatial context,^11,12^ and an extension of this research into the spatio-temporal domain is urgently required. This paper has shown that the development of more flexible localised smoothing models is likely to be beneficial in this regard, because locally smooth rather than globally smooth autocorrelation can be represented which is less likely to be collinear to globally smooth covariates.

The Glasgow study presented here is one of the most up to date studies of the long-term effects of air pollution on human health in a UK urban environment, and shows that even though pollution concentrations are relatively low they still represent a substantial public health problem. In particular, we have found that elevated concentrations of NO_2_ and PM_10_ are associated with increased risks of respiratory disease of 3.4% and 5.3% if the pollutants increased by 5.78 µgm^−3^ (NO_2_) and 1.96 µgm^−3^ (PM_10_), respectively. However, the limitations of data availability mean that we have assumed the impact of smoking can be accounted for by proxy measures of socio-economic deprivation, such as average property price and the proportion of people claiming JSA. Furthermore, the pollution data are modelled concentrations and thus subject to error, which could impact upon the analysis. Finally, this is a population level observational study, and the results must not be interpreted in terms of individual level cause and effect (ecological bias). Even so, as small-area studies are cheaper and quicker to implement than individual-level cohort studies, they form an important component of the evidence base quantifying the health effects of long-term exposure to air pollution.

There are many avenues for future work arising from this paper. The most obvious one from an epidemiological perspective would be to extend the present study to the whole of the United Kingdom, as it would give the UK government a national rather than a regional picture of the extent of the air pollution problem. As highlighted above there is also the problem of error in the modelled pollution concentrations, so an avenue of future work will be to combine the model considered here with a measurement error model for the pollution concentrations. Finally, the methodology proposed here would also be useful in the related field of disease mapping, whose aim is to estimate the spatio-temporal pattern in disease risk. Within this context our methodology would be especially suited to the identification of risk boundaries, which separate spatially or temporally adjacent data points that exhibit large differences in their disease risks. In this context, our approach would identify a set of disjoint risk boundaries that do not enclose a group of areal units and time points. Therefore, extending the algorithm so that it identifies closed boundaries that completely enclose a group of areal units and time points would be a useful tool in the cluster detection literature, where the identification of high-risk disease clusters is the main goal.

## Supplementary material

This paper is accompanied by supplementary material which includes details of a separable localised smoothing model and the data and software to partially reproduce the analysis in Section 6.
